# Supplementary material for: Structural basis of ligand binding modes at the human formyl peptide receptor 2
Source: Nat Commun. 2020 Mar 5;11:1208. doi: 10.1038/s41467-020-15009-1 (PMC7058083; doi:10.1038/s41467-020-15009-1)
Supplement: Supplementary file 1 — Supplementary Information [file 41467_2020_15009_MOESM1_ESM.pdf]

Structural basis of ligand binding modes at the human formyl peptide receptor 2

Supplementary Information

Tong Chen, Muya Xiong et al.

Supplementary Figures

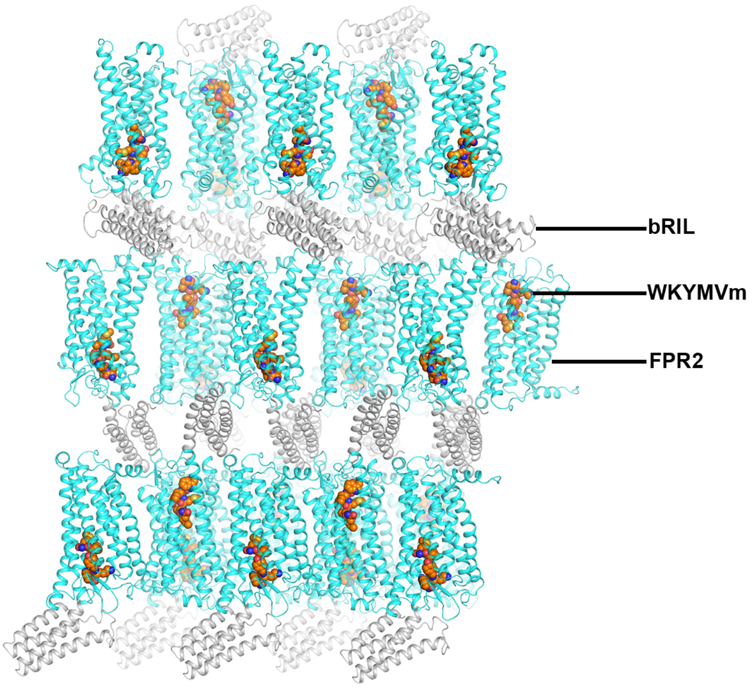

Supplementary Figure 1 | Crystal packing of FPR2-WKYMVm complex. FPR2 is shown in cartoon representation and colored cyan. The bRIL fusion protein is shown in grey cartoon representation. WKYMVm is displayed as orange spheres.

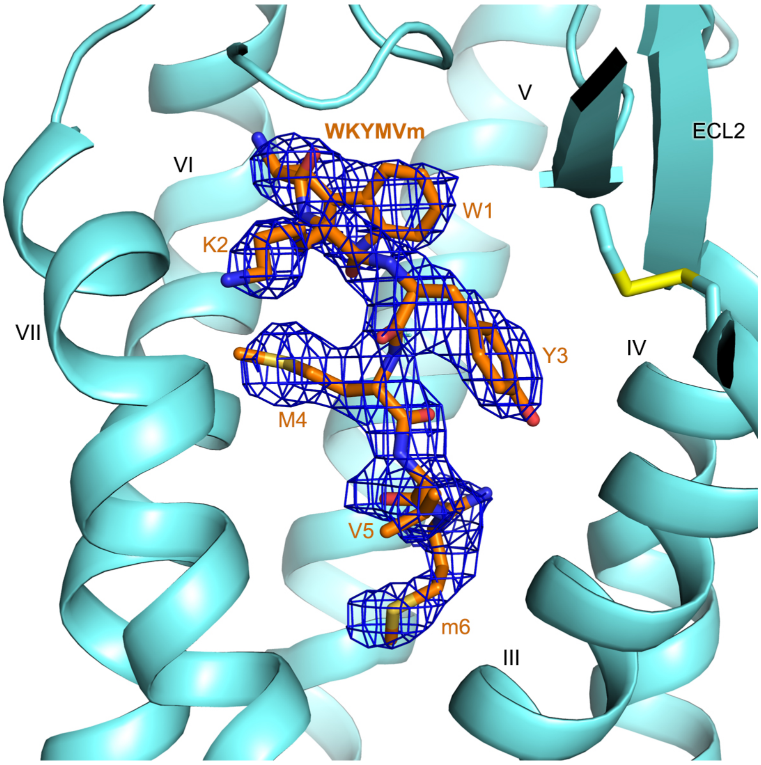

Supplementary Figure 2 | Electron densities of WKYMVm. The receptor is shown in cyan cartoon representation. The peptide WKYMVm is shown as orange sticks. The disulfide bond is shown as yellow sticks. Electron densities are contoured at 2.5σ from a $|F_o|$ - $|F_c|$ omit map and colored blue.

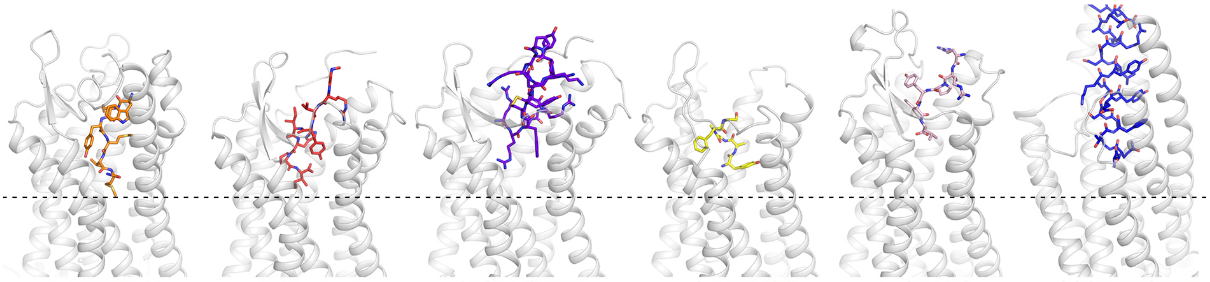

Supplementary Figure 3 | Comparison of the ligand-binding sites in peptide-bound GPCR structures. The receptors in the structures of FPR2-WKYMVm, AT2R-sAngII, CXCR4-CVX15, μOR-DAMGO, NTSR-NTS$_{8-13}$, and GCGR-NNC1702 are shown in cartoon representation and colored grey. The peptide ligands are colored orange (WKYMVm), red (sAngII), purple (CVX15), yellow (DAMGO), pink (NTS$_{8-13}$) and blue (NNC1702). The black dashed line indicates the deepest binding site that WKYMVm occupies within the receptor transmembrane helical bundle. The structures are displayed in a same orientation by aligning the other structures to the FPR2-WKYMVm structure.

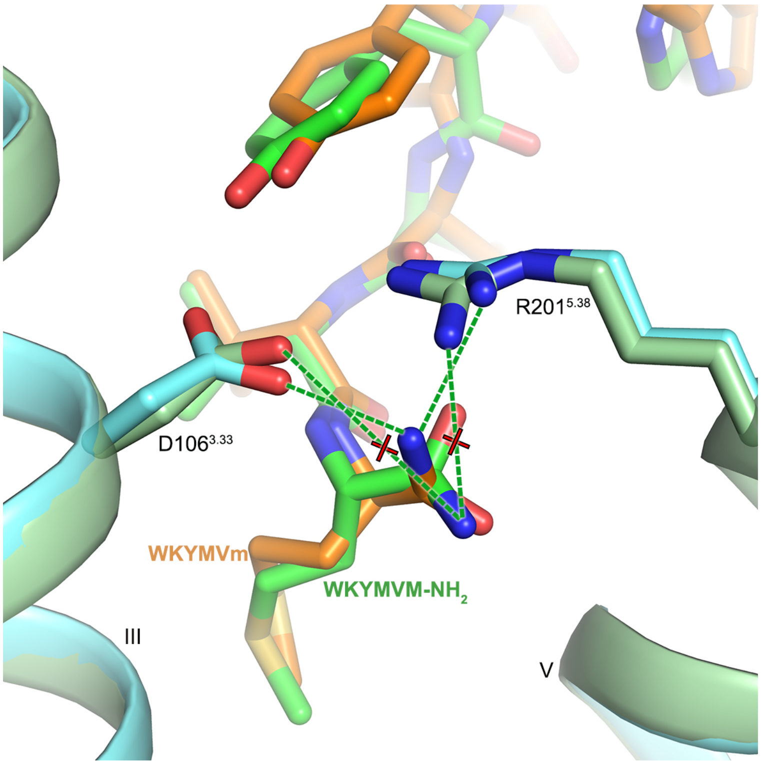

Supplementary Figure 4 | Molecular docking of WKYMVM-NH_2 in FPR2. The FPR2-WKYMVm crystal structure is colored cyan (FPR2) and orange (WKYMVm). The model of FPR2-WKYMVM-NH_2 is colored light green (FPR2) and green (WKYMVM-NH_2). The hydrogen bonds are shown as green dashed lines. The red 'x' indicates the break of the hydrogen bond between the C-terminal amide of the peptide and the receptor residue D106$^{3.33}$ or R201$^{5.38}$ in the WKYMVM-NH_2 docking model.

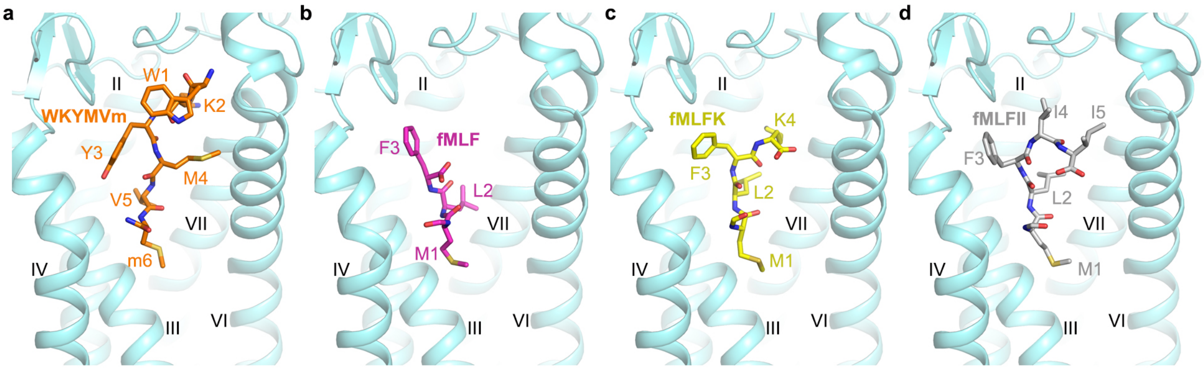

Supplementary Figure 5 | Comparison of binding sites for WKYMVm, fMLF, fMLFK and fMLFII in FPR2. **a** Crystal structure of FPR2-WKYMVm. **b** Docking model of FPR2-fMLF. **c** Docking model of FPR2-fMLFK. **d** Docking model of FPR2-fMLFII. The receptor in the crystal structure and models are shown in cyan cartoon representation. The ligands WKYMVm, fMLF, fMLFK and fMLFII are shown as sticks and colored orange, magenta, yellow and grey, respectively.

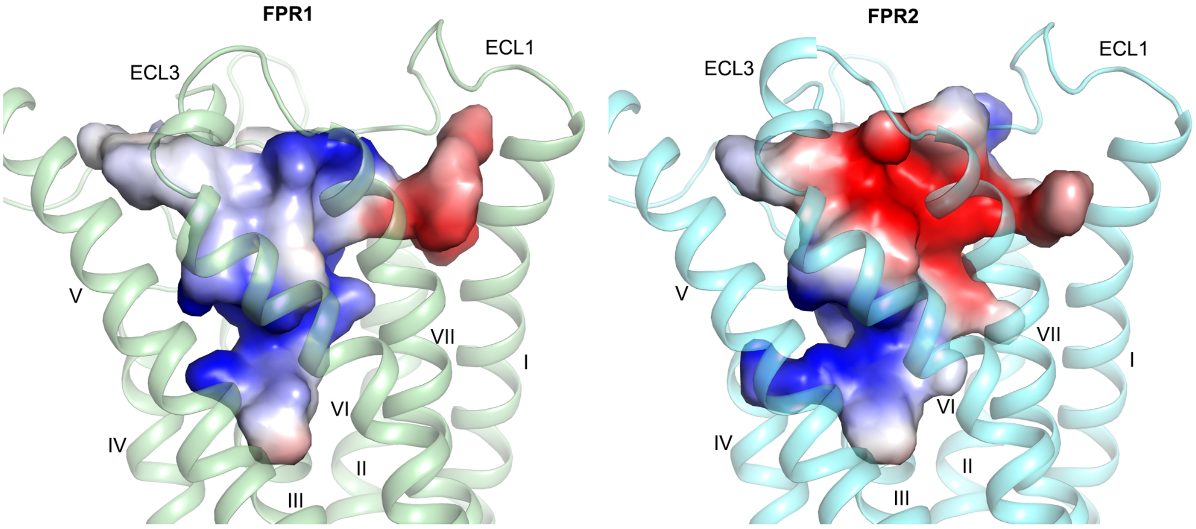

Supplementary Figure 6 | Ligand-binding pockets in FPR1 and FPR2. The receptors in the FPR1 model and FPR2-WKYMVm crystal structure are shown in cartoon representation and colored green (FPR1) and cyan (FPR2). Surfaces of ligand-binding pockets in both receptors are colored according to their electrostatic potential from red (negative) to blue (positive), showing different charge distribution within the ligand-binding pockets of these two receptors.

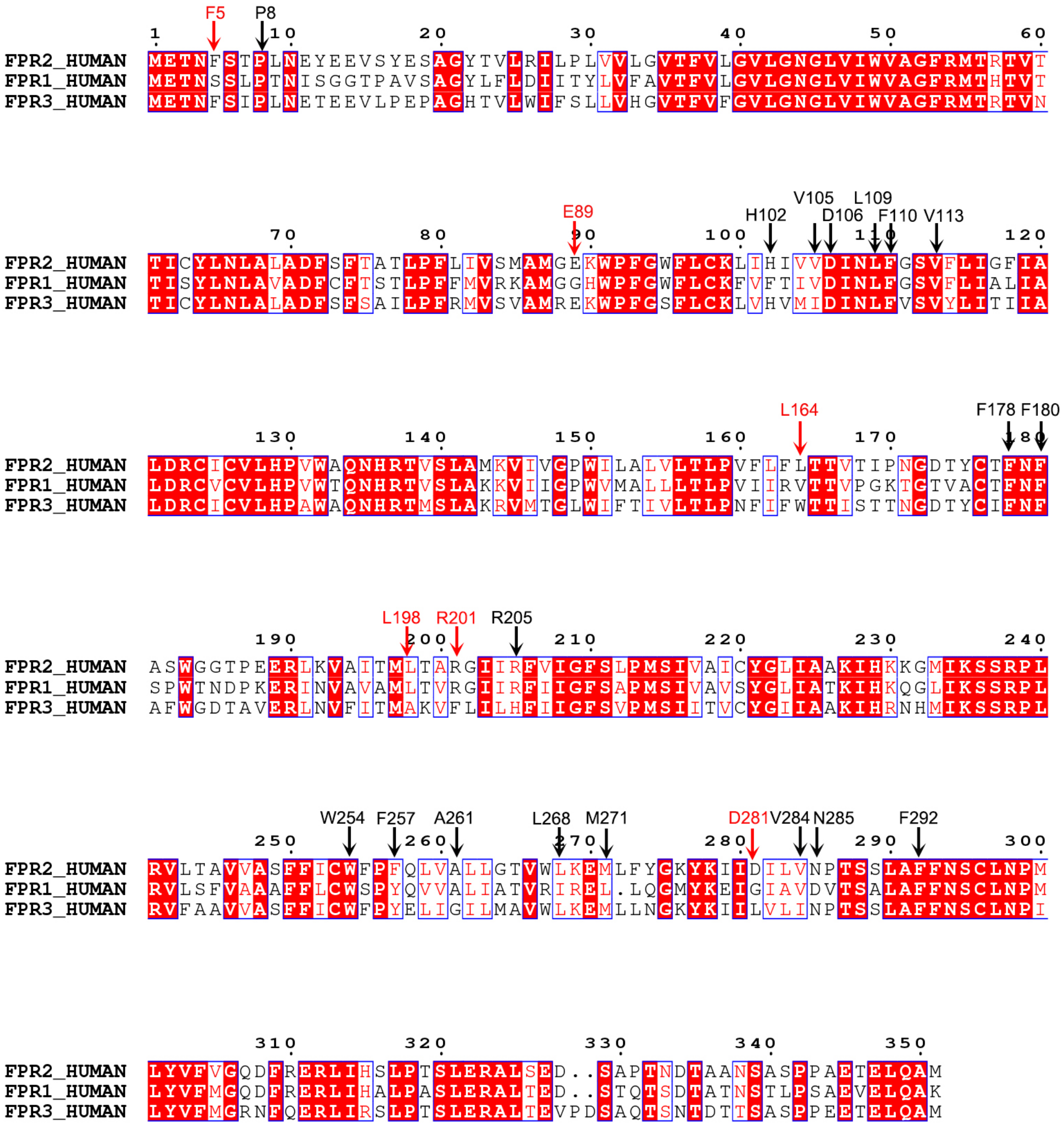

Supplementary Figure 7 | Sequence alignment of the human FPRs. Colors represent the similarity of residues: red background, identical; red text, strongly similar. Key residues in the WKYMVm-binding pocket, which are conserved or variable among receptors, are indicated by black or red arrows, respectively. The alignment was generated using UniProt (http://www.uniprot.org/align/) and the graphic was prepared on the ESPript 3.0 server (http://espript.ibcp.fr/ESPript/cgi-bin/ESPript.cgi).

Supplementary Table 1 | Binding of WK(FITC)YMVm to wild-type (WT) and mutant FPR2 and inhibition by fMLFK

WT/mutants	WK(FITC)YMVm, saturation binding			fMLFK, competition binding			Surface expression[d] (% of WT)
	K_d (nM)[a]	n[b]	Span[a,c] (% of WT)	K_i (μM)	pK_i [a]	n[b]	
WT	24.0 ± 8.5	3	100	83.1	4.08 ± 0.19	5	100
Construct[e]	27.7 ± 5.6	5	154 ± 21	/			126 ± 20
F5^{N}A[f]	15.1 ± 7.4	3	62 ± 7	/			92 ± 10
L81$^{2.60}$F	24.3 ± 8.4	3	42 ± 4	107	3.97 ± 0.27	4	93 ± 15
E89^{ECL1}G	29.3 ± 6.0	3	134 ± 7	322	3.49 ± 0.31	3	142 ± 22
H102$^{3.29}$A	ND	3	ND	/			76 ± 14
H102$^{3.29}$F	43.5 ± 16.0	3	44 ± 4	141	3.85 ± 0.24	3	43 ± 5
V105$^{3.32}$A	25.7 ± 7.5	4	68 ± 15	27.1	4.56 ± 0.21	3	124 ± 12
V105$^{3.32}$F	ND	3	ND	/			73 ± 10
D106$^{3.33}$A	ND	3	ND	/			22 ± 3
L109$^{3.36}$A	ND	3	ND	/			110 ± 11
F110$^{3.37}$A	ND	3	ND	/			42 ± 15
V113$^{3.40}$A	ND	3	ND	/			123 ± 17
V160$^{4.60}$A	36.1 ± 18.1	4	30 ± 4	68.7	4.16 ± 0.14	3	82 ± 6
L164^{ECL2}A	92.2 ± 25.1	3	85 ± 13	68.7	4.16 ± 0.25	3	69 ± 10
L164^{ECL2}W	ND	3	ND	/			87 ± 9
T177^{ECL2}A	109 ± 34	3	66 ± 5	108	3.97 ± 0.21	4	87 ± 8
F178^{ECL2}A	ND	3	ND	/			46 ± 11
F180^{ECL2}A	ND	3	ND	/			47 ± 8
L198$^{5.35}$A	41.5 ± 10.0	3	84 ± 14	/			84 ± 10
L198$^{5.35}$F	21.4 ± 5.3	3	60 ± 9	/			75 ± 8
R201$^{5.38}$A	ND	3	ND	/			37 ± 4
R201$^{5.38}$F	ND	4	ND	/			54 ± 11
R205$^{5.42}$A	ND	3	ND	/			30 ± 3
W254$^{6.48}$A	ND	3	ND	/			62 ± 11
F257$^{6.51}$A	ND	3	ND	/			100 ± 12
F257$^{6.51}$Y	154 ± 48	3	57 ± 6	/			82 ± 12
L268^{ECL3}A	28.0 ± 10.0	3	76 ± 7	38.2	4.42 ± 0.15	3	88 ± 7
M271^{ECL3}A	ND	3	ND	/			66 ± 8
D281$^{7.32}$A	48.6 ± 17.6	3	42 ± 8	/			89 ± 8
D281$^{7.32}$G	34.3 ± 14.1	4	39 ± 3	21.9	4.66 ± 0.16	3	85 ± 12
D281$^{7.32}$L	57.6 ± 14.0	3	39 ± 11	/			63 ± 9
V284$^{7.35}$A	ND	3	ND	/			64 ± 5
N285$^{7.36}$A	43.3 ± 15.3	4	85 ± 13	177	3.75 ± 0.16	3	108 ± 16
F292$^{7.43}$A	42.5 ± 14.7	3	54 ± 14	28.4	4.54 ± 0.11	3	80 ± 7

[a]Data are shown as mean ± s.e.m. from at least three independent experiments performed in

triplicate. Source data are provided as a Source Data file.

[b]Sample size; the number of independent experiments performed in triplicate.

[c]The span is defined as the window between the maximal WKYMVm response (E_{max}) and the vehicle (no ligand). ND (not determined) refers to data where a robust concentration response curve could not be established within the concentration range tested, such that an E_{max} was not reached and therefore span could not be calculated.

[d]Protein expression levels of FPR2 constructs at the cell surface were determined in parallel by flow cytometry with an anti-FLAG antibody and reported as per cent compared to the WT FPR2 from at least three independent measurements performed in duplicate.

[e]The FPR2 construct used for crystallization, containing the bRIL fusion at N terminus, the mutation S211$^{5.48}$L and 5 residues truncated at C terminus.

[f]All mutations were introduced to the WT FPR2.

Supplementary Table 2 | IP1 accumulation assays of wild-type (WT) and mutant FPR2s for the agonists WKYMVm and fMLFK using a chimeric Gα protein $G\alpha_{\Delta6qi4myr}$

WT/ mutants	WKYMVm				fMLFK				Surface expression[e] (% of WT)
	EC_{50}[a] (nM)	pEC_{50}[b]	n[c]	Span[b,d] (% of WT)	EC_{50}[a] (μM)	pEC_{50}[b]	n[c]	Span[b,d] (% of WT)	
WT	2.1	8.68 ± 0.08	14	100	4.9	5.31 ± 0.08	9	100	100
Construct[f]	1.7	8.77 ± 0.17	3	36 ± 8	/				126 ± 20
$F5^{N}A$[g]	1.7	8.76 ± 0.14	3	119 ± 16	/				92 ± 10
$L81^{2.60}F$	/				5.4	5.26 ± 0.26	3	120 ± 13	93 ± 15
$E89^{ECL1}A$	2.4	8.61 ± 0.19	3	67 ± 5	/				76 ± 3
$E89^{ECL1}G$	2.3	8.65 ± 0.23	3	91 ± 9	8.3	5.08 ± 0.20	3	120 ± 10	142 ± 22
$H102^{3.29}A$	15.5	7.81 ± 0.19	3	101 ± 13	16.0	4.79 ± 0.22	3	51 ± 5	76 ± 14
$H102^{3.29}F$	/				18.8	4.72 ± 0.19	3	93 ± 7	43 ± 5
$V105^{3.32}A$	213	6.67 ± 0.11	4	93 ± 12	0.84	6.08 ± 0.17	3	121 ± 9	124 ± 12
$V105^{3.32}F$	/				34.3	4.46 ± 0.15	3	65 ± 4	73 ± 10
$D106^{3.33}A$	ND	ND	3	ND	ND	ND	3	ND	22 ± 3
$D106^{3.33}L$	ND	ND	3	ND	/				16 ± 6
$L109^{3.36}A$	ND	ND	4	ND	ND	ND	3	ND	110 ± 11
$F110^{3.37}A$	24.9	7.60 ± 0.19	4	38 ± 17	2.7	5.58 ± 0.24	3	65 ± 7	42 ± 15
$V113^{3.40}A$	ND	ND	3	ND	ND	ND	3	ND	123 ± 17
$V160^{4.60}A$	/				6.7	5.17 ± 0.16	3	89 ± 6	82 ± 6
$L164^{ECL2}A$	210	6.68 ± 0.09	4	95 ± 4	49.2	4.31 ± 0.33	3	110 ± 30	69 ± 10
$L164^{ECL2}V$	1.1	8.98 ± 0.20	3	105 ± 12	/				82 ± 6
$L164^{ECL2}W$	ND	ND	4	ND	/				87 ± 9
$T177^{ECL2}A$	159	6.80 ± 0.13	3	130 ± 20	11.8	4.93 ± 0.18	3	140 ± 11	87 ± 8
$F178^{ECL2}A$	ND	ND	3	ND	ND	ND	3	ND	46 ± 11
$F180^{ECL2}A$	140	6.85 ± 0.09	5	82 ± 4	/				47 ± 8
$L198^{5.35}A$	137	6.86 ± 0.20	4	54 ± 5	/				84 ± 10
$L198^{5.35}F$	28.9	7.54 ± 0.15	3	117 ± 11	/				75 ± 8
$R201^{5.38}A$	195	6.71 ± 0.13	3	75 ± 5	ND	ND	3	ND	37 ± 4
$R201^{5.38}F$	ND	ND	4	ND	/				54 ± 11
$R205^{5.42}A$	ND	ND	4	ND	ND	ND	3	ND	30 ± 3
$S211^{5.48}L$	2.7	8.60 ± 0.17	5	66 ±7	/				118 ± 15
$W254^{6.48}A$	ND	ND	3	ND	ND	ND	3	ND	62 ± 11
$F257^{6.51}A$	ND	ND	3	ND	ND	ND	3	ND	100 ± 12
$L268^{ECL3}A$	0.45	9.35 ± 0.22	3	145 ± 13	3.1	5.51 ± 0.21	3	141 ± 11	88 ± 7
$M271^{ECL3}A$	2.1	8.67 ± 0.16	3	109 ± 24	4.1	5.39 ± 0.23	3	78 ± 7	66 ± 8
$M271^{ECL3}L$	3.0	8.52 ± 0.17	3	114 ± 16	/				64 ± 5
$D281^{7.32}A$	85.2	8.07 ± 0.15	4	96 ± 6	/				89 ± 8
$D281^{7.32}G$	157	6.80 ± 0.15	3	84 ± 6	1.7	5.77 ± 0.16	3	87 ± 6	85 ± 12
$D281^{7.32}L$	121	6.92 ± 0.18	3	82 ± 8	/				63 ± 9

V284$^{7.35}$A	/				12.3	4.91 ± 0.13	3	101 ± 6	64 ± 5
V284$^{7.35}$G	139	6.85 ± 0.07	4	99 ± 14	/				73 ± 8
V284$^{7.35}$F	/				600	3.22 ± 0.16	3	250 ± 44	84 ± 11
N285$^{7.36}$A	10.5	7.98 ± 0.17	3	85 ± 6	9.7	5.01 ± 0.09	3	98 ± 4	108 ± 16
F292$^{7.43}$A	7.5	8.12 ± 0.12	3	94 ± 6	1.0	5.00 ± 0.15	3	85 ± 5	80 ± 7

[a]EC_{50} values were determined after 1.5 h stimulation by increasing concentrations of WKYMVm or fMLFK.

[b]Data are shown as mean ± s.e.m. from at least three independent experiments performed in triplicate. Source data are provided as a Source Data file.

[c]Sample size; the number of independent experiments performed in triplicate.

[d]The span is defined as the window between the maximal WKYMVm/fMLFK response (E_{max}) and the vehicle (no ligand). ND (not determined) refers to data where a robust concentration response curve could not be established within the concentration range tested, such that an E_{max} was not reached and therefore span could not be calculated.

[e]Protein expression levels of FPR2 constructs at the cell surface were determined in parallel by flow cytometry with an anti-FLAG antibody and reported as per cent compared to the WT FPR2 from at least three independent measurements performed in duplicate.

[f]The FPR2 construct used for crystallization, containing the bRIL fusion at N terminus, the mutation S211$^{5.48}$L and 5 residues truncated at C terminus.

[g]All mutations were introduced to the WT FPR2.

Supplementary Table 3 | Data collection and refinement statistics

	FPR2-WKYMVm
**Data Collection**[a]	
Space group	$P3_221$
Cell dimensions	
a,b,c (Å)	66.3, 66.3, 244.9
α,β,γ (°)	90.0, 90.0, 120.0
Resolution (Å)	30.0-2.8 (2.9-2.8)[b]
R_{merge}	0.14 (0.71)
$I / \sigma(I)$	6.8 (1.1)
$CC_{1/2}$	0.99 (0.79)
Completeness (%)	96.5 (94.3)
Redundancy	7.9 (6.3)
**Refinement**	
Resolution (Å)	30.0-2.8
No. reflections	15,644
R_{work} / R_{free}	0.258 / 0.289
No. atoms	
Protein	3,337
Ligand	59
B-factors (Å^2)	
Protein	106
Ligand	90
R.m.s deviations	
Bond lengths (Å)	0.002
Bond angles (°)	0.45

[a]Diffraction data from 28 FPR2-WKYMVm crystals were used to solve the structure.
[b]Values in parentheses are for highest-resolution shell.

Supplementary Table 4 | Codon-optimized DNA sequence of FPR2 and primer sequences

FPR2-WT DNA sequence
GGCGCGCCATGGAAACCAACTTCTCAACCCCGCTGAACGAATACGAGGAGGTCTCATA<br>CGAATCCGCAGGCTACACGGTGCTCCGCATCTTGCCCTTGGTGGTCCTGGGCGTCACCT<br>TCGTTCTCGGAGTGTTGGGAAACGGTCTGGTCATCTGGGTTGCCGGTTTCAGGATGACC<br>AGAACTGTTACCACTATTTGCTACTTGAACCTGGCTCTCGCCGACTTCTCCTTCACAGCT<br>ACGCTGCCTTTCCTCATCGTGAGCATGGCCATGGGAGAGAAGTGGCCCTTCGGCTGGTT<br>CTTGTGTAAACTGATCCACATTGTTGTGGACATCAACTTGTTCGGATCTGTCTTCCTGAT<br>CGGTTTCATTGCTCTCGATCGCTGCATTTGTGTGCTGCACCCAGTCTGGGCACAGAACC<br>ATCGTACAGTGTCACTGGCGATGAAGGTCATCGTCGGCCCATGGATTTTGGCACTGGTT<br>CTCACATTGCCGGTGTTCCTGTTCCTCACAACGGTCACGATCCCAAACGGAGATACCTA<br>CTGCACTTTCAACTTCGCCTCCTGGGGTGGCACTCCGGAGGAAAGGTTGAAGGTGGCA<br>ATCACAATGCTGACGGCGAGGGGCATCATTAGATTCGTGATTGGATTCTCTCTGCCTATG<br>TCAATCGTCGCTATTTGCTACGGTCTCATCGCTGCCAAGATTCACAAGAAAGGCATGATC<br>AAATCCAGCCGCCCTCTCCGTGTCTTGACCGCAGTCGTTGCGAGTTTCTTCATCTGTTG<br>GTTCCCCTTCCAACTGGTCGCTCTGCTCGGAACTGTTTGGCTGAAGGAGATGCTCTTCT<br>ACGGCAAGTACAAAATCATTGACATCCTCGTGAACCCAACTTCTTCATTGGCTTTCTTCA<br>ACTCTTGTTTGAACCCGATGCTGTACGTTTTCGTGGGCCAGGATTTCCGCGAACGTCTC<br>ATCCATAGTTTGCCTACATCGCTGGAGAGAGCTCTCTCCGAAGACAGCGCACCAACCAA<br>CGATACTGCAGCGAACAGTGCTTCGCCTCCCGCCGAGACGGAACTGCAAGCCATGGAA<br>TTC

Primer sequences	
bRIL-F	ATTGGCGCGCCGGCTGATCTGGAAGACAATTGGGAA
bRIL-FPR2-F	ATTGGCGCGCCGACCAACTTCTCAACCCCG
C-truncation-R	CCGGAATTCCGTCTCGGCGGGAGGCGAAGCACTGTT
F5A-F	ATTGGCGCGCCGGAAACCAACGCATCAACCCCGCTGAACGAATAC
F5A-R	GTATTCGTTCAGCGGGGTTGATGCGTTGGTTTCCGGCGCGCCAAT
L81F-F	TTCACAGCTACGCTGCCTTTCTTCATCGTGAGCATGGCCATGGGA
L81F-R	TCCCATGGCCATGCTCACGATGAAGAAAGGCAGCGTAGCTGTGAA
E89A-F	TCATCGTGAGCATGGCCATGGGAGCAAAGTGGCCCTTCGGCTGGT
E89A-R	ACCAGCCGAAGGGCCACTTTGCTCCCATGGCCATGCTCACGATGA
E89G-F	ATCGTGAGCATGGCCATGGGAGGCAAGTGGCCCTTCGGCTGGTTC
E89G-R	GAACCAGCCGAAGGGCCACTTGCCTCCCATGGCCATGCTCACGAT
H102A-F	TTCTTGTGTAAACTGATCGCCATTGTTGTGGACATCAACTTGTTC
H102A-R	GAACAAGTTGATGTCCACAACAATGGCGATCAGTTTACACAAGAA
H102F-F	TGGTTCTTGTGTAAACTGATCTTCATTGTTGTGGACATCAACTTG
H102F-R	CAAGTTGATGTCCACAACAATGAAGATCAGTTTACACAAGAACCA
V105A-F	TGTAAACTGATCCACATTGTTGCCGACATCAACTTGTTCGGATCT
V105A-R	AGATCCGAACAAGTTGATGTCGGCAACAATGTGGATCAGTTTACA
V105F-F	GTAAACTGATCCACATTGTTTTCGACATCAACTTGTTCGGATCT
V105F-R	AGATCCGAACAAGTTGATGTCGAAAACAATGTGGATCAGTTTACA
D106A-F	TAAACTGATCCACATTGTTGTGGCCATCAACTTGTTCGGATCTGT
D106A-R	ACAGATCCGAACAAGTTGATGGCCACAACAATGTGGATCAGTTTA

D106L-F	GACAGATCCGAACAAGTTGATCAACACAACAATGTGGATCAGTTT
D106L-R	AAACTGATCCACATTGTTGTGTTGATCAACTTGTTCGGATCTGTC
L109A-F	CACATTGTTGTGGACATCAACGCCTTCGGATCTGTCTTCCTGATC
L109A-R	GATCAGGAAGACAGATCCGAAGGCGTTGATGTCCACAACAATGTG
F110A-F	CATTGTTGTGGACATCAACTTGGCAGGATCTGTCTTCCTGATCGG
F110A-R	CCGATCAGGAAGACAGATCCTGCCAAGTTGATGTCCACAACAATG
V113A-F	GACATCAACTTGTTCGGATCTGCTTTCCTGATCGGTTTCATTGCT
V113A-R	AGCAATGAAACCGATCAGGAAAGCAGATCCGAACAAGTTGATGTC
V160A-F	GCACTGGTTCTCACATTGCCGGCATTCCTGTTCCTCACAACGGTC
V160A-R	GACCGTTGTGAGGAACAGGAATGCCGGCAATGTGAGAACCAGTGC
L164A-F	CATTGCCGGTGTTCCTGTTCGCAACAACGGTCACGATCCCAAAC
L164A-R	GTTTGGGATCGTGACCGTTGTTGCGAACAGGAACACCGGCAATG
L164V-F	ACATTGCCGGTGTTCCTGTTCGTCACAACGGTCACGATCCCAAAC
L164V-R	GTTTGGGATCGTGACCGTTGTGACGAACAGGAACACCGGCAATGT
L164W-F	ACATTGCCGGTGTTCCTGTTCTGGACAACGGTCACGATCCCAAAC
L164W-R	GTTTGGGATCGTGACCGTTGTCCAGAACAGGAACACCGGCAATGT
T177A-F	CCAAACGGAGATACCTACTGCGCATTCAACTTCGCCTCCTGGGGT
T177A-R	ACCCCAGGAGGCGAAGTTGAATGCGCAGTAGGTATCTCCGTTTGG
F178A-F	AACGGAGATACCTACTGCACTGCAAACTTCGCCTCCTGGGGTGGC
F178A-R	GCCACCCCAGGAGGCGAAGTTTGCAGTGCAGTAGGTATCTCCGTT
F180A-F	GATACCTACTGCACTTTCAACGCAGCCTCCTGGGGTGGCACTCCG
F180A-R	CGGAGTGCCACCCCAGGAGGCTGCGTTGAAAGTGCAGTAGGTATC
L198A-F	TTGAAGGTGGCAATCACAATGGCCACGGCGAGGGGCATCATTAGA
L198A-R	TCTAATGATGCCCCTCGCCGTGGCCATTGTGATTGCCACCTTCAA
L198F-F	TTGAAGGTGGCAATCACAATGTTCACGGCGAGGGGCATCATTAGA
L198F-R	TCTAATGATGCCCCTCGCCGTGAACATTGTGATTGCCACCTTCAA
R201A-F	GCAATCACAATGCTGACGGCGGCAGGCATCATTAGATTCGTGATT
R201A-R	AATCACGAATCTAATGATGCCTGCCGCCGTCAGCATTGTGATTGC
R201F-F	GCAATCACAATGCTGACGGCGTTCGGCATCATTAGATTCGTGATT
R201F-R	AATCACGAATCTAATGATGCCGAACGCCGTCAGCATTGTGATTGC
R205A-F	CTGACGGCGAGGGGCATCATTGCCTTCGTGATTGGATTCTCTCTG
R205A-R	CAGAGAGAATCCAATCACGAAGGCAATGATGCCCCTCGCCGTCAG
S211L-F	TAGATTCGTGATTGGATTCCTGCTGCCTATGTCAATCGTCGCTAT
S211L-R	ATAGCGACGATTGACATAGGCAGCAGGAATCCAATCACGAATCTA
W254A-F	GTTGCGAGTTTCTTCATCTGTGCCTTCCCCTTCCAACTGGTCGCT
W254A-R	AGCGACCAGTTGGAAGGGGAAGGCACAGATGAAGAAACTCGCAAC
F257A-F	GTTTCTTCATCTGTTGGTTCCCCGCCCAACTGGTCGCTCTGCTCG
F257A-R	CGAGCAGAGCGACCAGTTGGGCGGGGAACCAACAGATGAAGAAAC
F257Y-F	TTCTTCATCTGTTGGTTCCCCTACCAACTGGTCGCTCTGCTCGGA
F257Y-R	TCCGAGCAGAGCGACCAGTTGGTAGGGGAACCAACAGATGAAGAA
L268A-F	GCTCTGCTCGGAACTGTTTGGGCTAAGGAGATGCTCTTCTACGGC
L268A-R	GCCGTAGAAGAGCATCTCCTTAGCCCAAACAGTTCCGAGCAGAGC
M271A-F	GGAACTGTTTGGCTGAAGGAGGCTCTCTTCTACGGCAAGTACAAA
M271A-R	TTTGTACTTGCCGTAGAAGAGAGCCTCCTTCAGCCAAACAGTTCC

M271L-F	GGAACTGTTTGGCTGAAGGAGCTCCTCTTCTACGGCAAGTACAAA
M271L-R	TTTGTACTTGCCGTAGAAGAGGAGCTCCTTCAGCCAAACAGTTCC
D281A-F	TACGGCAAGTACAAAATCATTGCAATCCTCGTGAACCCAACTTCT
D281A-R	AGAAGTTGGGTTCACGAGGATTGCAATGATTTTGTACTTGCCGTA
D281G-F	ACGGCAAGTACAAAATCATTGGAATCCTCGTGAACCCAACTTCT
D281G-R	AGAAGTTGGGTTCACGAGGATTCCAATGATTTTGTACTTGCCGTA
D281L-F	TACGGCAAGTACAAAATCATTCTGATCCTCGTGAACCCAACTTCT
D281L-R	AGAAGTTGGGTTCACGAGGATCAGAATGATTTTGTACTTGCCGTA
V284A-F	TACAAAATCATTGACATCCTCGCTAACCCAACTTCTTCATTGGCT
V284A-R	AGCCAATGAAGAAGTTGGGTTAGCGAGGATGTCAATGATTTTGTA
V284G-F	TACAAAATCATTGACATCCTCGGAAACCCAACTTCTTCATTGGCT
V284G-R	AGCCAATGAAGAAGTTGGGTTTCCGAGGATGTCAATGATTTTGTA
V284F-F	TACAAAATCATTGACATCCTCTTCAACCCAACTTCTTCATTGGCT
V284F-R	AGCCAATGAAGAAGTTGGGTTGAAGAGGATGTCAATGATTTTGTA
N285A-F	AAAATCATTGACATCCTCGTGGCACCAACTTCTTCATTGGCTTTC
N285A-R	GAAAGCCAATGAAGAAGTTGGTGCCACGAGGATGTCAATGATTTT
F292A-F	AACCCAACTTCTTCATTGGCTGCTTTCAACTCTTGTTTGAACCCG
F292A-R	CGGGTTCAAACAAGAGTTGAAAGCAGCCAATGAAGAAGTTGGGTT
