# Supplementary material for: Structural basis of ligand binding modes at the human formyl peptide receptor 2
Source: Nat Commun. 2020 Mar 5;11:1208. doi: 10.1038/s41467-020-15009-1 (PMC7058083; doi:10.1038/s41467-020-15009-1)
Supplement: Supplementary file 3 — Description of Additional Supplementary Information [file 41467_2020_15009_MOESM3_ESM.pdf]

Description of Additional Supplementary Files

File Name: Supplementary Data 1
Description: Molecular docking model of FPR2-WKYMVM

File Name: Supplementary Data 2
Description: Molecular docking model of FPR2-fMLF

File Name: Supplementary Data 3
Description: Molecular docking model of FPR1-fMLF

File Name: Supplementary Data 4
Description: Molecular docking model of FPR2- fMLFK

File Name: Supplementary Data 5
Description: Molecular docking model of FPR2-fMLFII
