# Supplementary material for: Structural basis of ligand binding modes at the human formyl peptide receptor 2
Source: Nat Commun. 2020 Mar 5;11:1208. doi: 10.1038/s41467-020-15009-1 (PMC7058083; doi:10.1038/s41467-020-15009-1)
Supplement: Supplementary file 9 — Reporting Summary [file 41467_2020_15009_MOESM9_ESM.pdf]

nature research

Corresponding author(s): Beili Wu, Qiang Zhao and Yechun Xu

Last updated by author(s): Feb 7, 2020

Reporting Summary

Nature Research wishes to improve the reproducibility of the work that we publish. This form provides structure for consistency and transparency in reporting. For further information on Nature Research policies, see Authors & Referees and the Editorial Policy Checklist .

Statistics

For all statistical analyses, confirm that the following items are present in the figure legend, table legend, main text, or Methods section.

n/a	Confirmed	
☐	☒	The exact sample size (*n*) for each experimental group/condition, given as a discrete number and unit of measurement
☐	☒	A statement on whether measurements were taken from distinct samples or whether the same sample was measured repeatedly
☒	☐	The statistical test(s) used AND whether they are one- or two-sided<br>*Only common tests should be described solely by name; describe more complex techniques in the Methods section.*
☒	☐	A description of all covariates tested
☒	☐	A description of any assumptions or corrections, such as tests of normality and adjustment for multiple comparisons
☐	☒	A full description of the statistical parameters including central tendency (e.g. means) or other basic estimates (e.g. regression coefficient) AND variation (e.g. standard deviation) or associated estimates of uncertainty (e.g. confidence intervals)
☒	☐	For null hypothesis testing, the test statistic (e.g. *F*, *t*, *r*) with confidence intervals, effect sizes, degrees of freedom and *P* value noted<br>*Give P values as exact values whenever suitable.*
☒	☐	For Bayesian analysis, information on the choice of priors and Markov chain Monte Carlo settings
☒	☐	For hierarchical and complex designs, identification of the appropriate level for tests and full reporting of outcomes
☒	☐	Estimates of effect sizes (e.g. Cohen's *d*, Pearson's *r*), indicating how they were calculated

Our web collection on statistics for biologists contains articles on many of the points above.

Software and code

Policy information about availability of computer code

Data collection	No commercial and open source software was used for data collection.
Data analysis	The following softwares were used in X-ray diffraction data processing and model building: XDS, Phaser, PHENIX and Buster.<br>The signaling and ligand binding data were analysed by GraphPad Prism 7.0.<br>The softwares used in molecular docking: Schrödinger suite.

For manuscripts utilizing custom algorithms or software that are central to the research but not yet described in published literature, software must be made available to editors/reviewers. We strongly encourage code deposition in a community repository (e.g. GitHub). See the Nature Research guidelines for submitting code & software for further information.

Data

Policy information about availability of data

All manuscripts must include a data availability statement. This statement should provide the following information, where applicable:

- Accession codes, unique identifiers, or web links for publicly available datasets
- A list of figures that have associated raw data
- A description of any restrictions on data availability

Atomic coordinates and structure factors of the FPR2-WKYMVm structure have been deposited in the Protein Data Bank with accession code 6LW5. The source data underlying Fig. 4 and Supplementary Tables 1 and 2 are provided as a Source Data file. Other data are available from the corresponding authors upon reasonable request.

Field-specific reporting

Please select the one below that is the best fit for your research. If you are not sure, read the appropriate sections before making your selection.

☒ Life sciences ☐ Behavioural & social sciences ☐ Ecological, evolutionary & environmental sciences

For a reference copy of the document with all sections, see nature.com/documents/nr-reporting-summary-flat.pdf

Life sciences study design

All studies must disclose on these points even when the disclosure is negative.

Sample size	Due to radiation damage, X-ray diffraction data collection of the protein crystals was limited to 5-10 degree per crystal. To collect a complete data set for structure determination, diffraction data from multiple crystals were integrated and scaled using XDS. By calculating completeness of the data set, diffraction data from 28 FPR2-WKYMVm crystals were used to ensure the completeness was close to 100%. For ligand binding and IP accumulation assays, at least 3 independent experiments were performed in triplicate to ensure each data point was repeatable.
Data exclusions	No data were excluded from the analyses.
Replication	All attempts at replication were successful.
Randomization	Randomization is not relevant to this study, as protein and crystal samples are not required to be allocated into experimental groups in protein structural studies, and no animals or human research participants are involved in this study.
Blinding	Blinding is not relevant to this study, as protein and crystal samples are not required to be allocated into experimental groups in protein structural studies, and no animals or human research participants are involved in this study.

Reporting for specific materials, systems and methods

We require information from authors about some types of materials, experimental systems and methods used in many studies. Here, indicate whether each material, system or method listed is relevant to your study. If you are not sure if a list item applies to your research, read the appropriate section before selecting a response.

Materials & experimental systems

n/a	Involved in the study	
☐	☒	Antibodies
☐	☒	Eukaryotic cell lines
☒	☐	Palaeontology
☒	☐	Animals and other organisms
☒	☐	Human research participants
☒	☐	Clinical data

Methods

n/a	Involved in the study	
☒	☐	ChIP-seq
☒	☐	Flow cytometry
☒	☐	MRI-based neuroimaging

Antibodies

Antibodies used	Cryptate-labeled anti-IP1 monoclonal antibody: CisBio Bioassays, Cat#62IPAPEB.<br>Monoclonal ANTI-FLAG M2-FITC antibody: Sigma, F4049; 1:100 diluted by TBS supplemented with 4% BSA.
Validation	The antibodies have been commercially obtained and are described as required in Methods.

Eukaryotic cell lines

Policy information about cell lines

Cell line source(s)	The HEK293F and HEK293 cell lines were originally obtained from Invitrogen.
Authentication	None of the cell lines have been authenticated.
Mycoplasma contamination	The cell lines were negative for mycoplasma contamination.
Commonly misidentified lines<br>(See ICLAC register)	No commonly misidentified cell lines were used.
